# Recovery of Oculomotor Nerve Palsy After Treatment of Posterior Communicating Artery Aneurysms: Have the Outcomes Changed?

**DOI:** 10.7759/cureus.95958

**Published:** 2025-11-02

**Authors:** Neha Ramu, Swati Jain, Tamara Tajsic, Samir Matloob, Daniel Brown, Mathew Guilfoyle, Adel Helmy, Rikin Trivedi

**Affiliations:** 1 Neurology, Guy's and St Thomas' NHS Foundation Trust, London, GBR; 2 Neuroscience, University of Cambridge, Cambridge, GBR; 3 Neurosurgery, Addenbrooke's Hospital, Cambridge University Hospitals NHS Foundation Trust, Cambridge, GBR; 4 Neurosurgery, Oxford University Hospitals NHS Foundation Trust, Oxford, GBR

**Keywords:** aneurysmal subarachnoid haemorrhage, endovascular coiling, oculomotor nerve palsy, posterior communicating artery aneurysm, surgical clipping

## Abstract

Introduction

It is unclear whether microsurgical clipping or endovascular coiling is the best treatment for recovery from oculomotor nerve palsy (ONP) secondary to posterior communicating artery (PComm) aneurysm. This is a five-year study of aneurysms presenting with ONP and their outcomes after treatment.

Method

The study included all patients from 2017-2022 who presented to a tertiary care centre in the United Kingdom with complete or partial ONP from ruptured or unruptured PComm aneurysms. Electronic medical records of these patients were compared with data from the same unit of an earlier cohort of patients.

Results

A total of 165 patients with PComm aneurysms were identified in the five-year period. Of these, 30 presented with ONP, of which 17 were complete. A total of 20 patients presented with a subarachnoid haemorrhage; 10 patients underwent microsurgical clipping, and 40% of them had improved ONPs at six months, whereas only 20% of the coiling group improved. The odds ratio of full recovery of ONP with coiling was 0.20 (0.03-1.07) when compared to clipping. Overall, these results were largely similar to those of the earlier cohort; however, this study noted a difference in the rate of recovery depending on treatment modality, albeit not statistically significant.

Conclusion

No significant difference in rates of recovery of ONPs was found between surgical clipping or endovascular coiling, although this conclusion is limited by the small sample size. Both modalities continue to appear equally effective for aneurysm management.

## Introduction

Posterior communicating artery (PComm) aneurysms comprise 25% of all intracranial aneurysms and can arise anywhere along the PComm artery, from the junction with the internal carotid to the junction with the posterior cerebral artery [1]. PComm aneurysms can present as unruptured or ruptured aneurysms with subarachnoid haemorrhages (SAH) [2]. Both presentations can result in substantial clinical manifestations [3-5], with 30-50% of patients developing oculomotor nerve palsy (ONP) due to its intimate anatomical relationship with the PComm [6].

ONPs due to unruptured aneurysms can be attributed to aneurysmal sac pulsatility and expansion, impending rupture, or venous congestion; when accompanying SAH, ONPs are often due to direct compression of the cisternal segment of the nerve due to rapid expansion in size of the aneurysm or irritation/injury to the nerve from the SAH [7,8]. Complete ONPs typically present with ptosis, ophthalmoplegia, and anisocoric mydriasis, whereas partial ONPs have one or two of these symptoms [9].

Treatment of PComm aneurysms can be accomplished by both microsurgical clipping and endovascular coiling, and the chosen modality depends on a number of anatomical and clinical factors [2]. It remains contentious if either of the two treatments provides a therapeutic advantage where recovery of ONP is concerned in the presence of PComm aneurysm. Microsurgical clipping allows direct access to remove the mass effect of the aneurysm on the oculomotor nerve, postulated to allow improved chances of recovery [10]. Endovascular treatment provides an alternative and less invasive method [11,12]; however, it is theorised that the inability to remove the mass effect and additional direct effect of the coils in the aneurysm on the nerve may lead to poorer outcomes where ONP recovery is concerned.

Various studies have offered mixed results [13-15]. Recent studies by interventional radiology groups have demonstrated reasonable improvement of ONPs after endovascular treatment [11,12]. A meta-analysis by Gaberel et al. in 2016 evaluated 11 studies with 384 patients who presented with ONPs and found that 83.6% of patients who underwent clipping had complete recovery compared to 42.5% in the endovascular group [10].

A previous study by Patel et al. was conducted over a five-year period from 2005 to 2009 at the same centre as this current study [16]. It evaluated the outcomes between endovascular treatment and surgical clipping in a small cohort of patients and did not find any significant difference in ONP recovery dependent on the modality of treatment. Almost two decades later, this study is being carried out to understand if advancements in endovascular treatments, early diagnosis, increased findings of incidental aneurysms, etc., have had any impact on the recovery of ONPs. This study aims to evaluate how the choice of treatment modality (clipping versus coiling) influences the rate of recovery of ONPs in patients with PComm aneurysms over a recent five-year period.

## Materials and methods

This was a retrospective study conducted at a single tertiary neurosurgical centre, Addenbrooke's Hospital, Cambridge University Hospitals NHS Foundation Trust, Cambridge, United Kingdom. Anonymised data for the study were collected retrospectively from 2017 to 2022 from an existing neurovascular database, which included all patients who had presented with internal carotid artery, PComm, and posterior cerebral artery aneurysms.

The inclusion criteria were all the patients who presented with a PComm aneurysm and also had an ONP. Patients were included regardless of demographics, diagnosis, size of aneurysm, severity of ONP, or outcome of hospital admission, in order to encapsulate as broad a sample as possible in this niche area. Exclusion criteria were limited to patients in whom the ONP preceded the Pcomm due to a different cause, those who did not undergo any intervention, or those who died prior to assessment of the ONP on admission. 

Standard demographic data, including age, gender, nature of presentation, ruptured versus unruptured aneurysm, and treatment modality, were all collected from the neurovascular database, which is a subset of records extracted from the larger Epic® electronic healthcare system database (Epic Systems Corporation, Verona, Wisconsin, United States). Where available, data from follow-up ophthalmology assessments were also collected. As patients with acute onset ONP may have had a sentinel bleed, patients were said to have an incidental PComm aneurysm if there was no radiological evidence of subarachnoid haemorrhage and a negative lumbar puncture (no xanthochromia or bilirubin) when presenting with acute ONP.

Decision for surgical clipping or endovascular coiling was determined after discussion between the consultant neurovascular neurosurgeon and interventional neuroradiologist, based on the patients' clinical presentation and anatomical considerations. Patients who presented with acute ruptured aneurysms received definitive treatment within 24 hours of referral to our neurosurgical centre. During surgical clipping, the surgeon could choose to decompress the sac if considered safe. No attempts were made to separate the aneurysmal sac from the oculomotor nerve. Any immediate or delayed post-operative complications were recorded as part of the data collection for both treatment modalities.

Classification of ONPs

Patients were classified as having complete ONPs if they had all three findings of ptosis, ophthalmoplegia, and mydriasis on presentation, or partial ONP if only one or two of the findings were present. Improvement of ONP was defined as resolution of one or more of the above symptoms, with full recovery being the resolution of all three. All patients were followed up for at least six months. A follow-up assessment for all patients was performed in 2022 to document the recovery of patients’ ONP after treatment. Due to changes in how patients were reviewed in the outpatient clinic during COVID-19, the follow-up was often limited to telephone consultations between 2021 and 2022. The written documentation of telephone consultations or letters from their general practitioner (GP) was reviewed to document the recovery of ONP in these patients. If patients were lost to follow-up or died prior to discharge, the last assessment of ONP was recorded.

Data analysis

Using R (R Foundation for Statistical Computing, Vienna, Austria, https://www.R-project.org/), statistical tests, such as logistic regression and Chi-square, were applied to calculate p-values and odds ratios.

## Results

Demographics and treatment modalities

Between January 2017 and August 2022, 165 patients with PComm aneurysms were identified, of which 30 (18.2%) had ONP. There were 22 female patients, and the mean age was 58.1 ± 13.7 years (range: 36-85 years). Table 1 describes the detailed demographic characteristics of the cohort.

**Table 1:** Demographic details of patients who presented with ONP in presence of PComm aneurysm (N=30) ONP: oculomotor nerve palsy; Pcomm: posterior communicating artery

Variables	Clipping (n=10)	Coiling (n-20)	Total (n-30)	P value
Age (years), mean	51.5	61.4	58.1	0.06
Male, n	2	6	8	0.16
Female, n	8	14	22	0.2
Ruptured, n	7	13	20	0.18
Unruptured, n	3	7	10	0.21
Left, n	8	3	11	0.13
Right, n	2	17	19	<0.05
Aneurysm size (mm), mean	9.6	6.9	7.8	0.11
Length of stay (days), mean	12.0	14.0	13.5	0.66

A total of 20 patients presented with SAH features (consistent with a ruptured PComm aneurysm), with the majority (n=19, 63.3%) having a right-sided PComm aneurysm. Of the patients presenting with acute SAH, 75% (n=15) were World Federation of Neurosurgical Societies (WFNS) grade 1 and 2. The size of the aneurysm in the ruptured group was higher than that of the unruptured group, although not statistically significant (8.42 mm vs 6.25 mm, p value = 0.21).

A total of 20 (60%) patients underwent endovascular coiling, whilst 10 underwent microsurgical clipping. The mean age of those who underwent coiling was higher than those who underwent clipping (61.4 ± 13.9 vs 51.5 ± 11.1 years, p value = 0.06). Right-sided PComm aneurysms were significantly (*p*=0.0006) more likely to undergo coiling. The size of the aneurysm was higher in the clipping group as compared to the coiling group (9.56 mm vs 6.98 mm, p value = 0.11). Of the 20 patients who presented with acute SAH, the majority were coiled (n=13, 65%). Of the 10 patients who presented with unruptured aneurysms, seven patients underwent endovascular treatment.

ONP presentation

**Figure 1:**
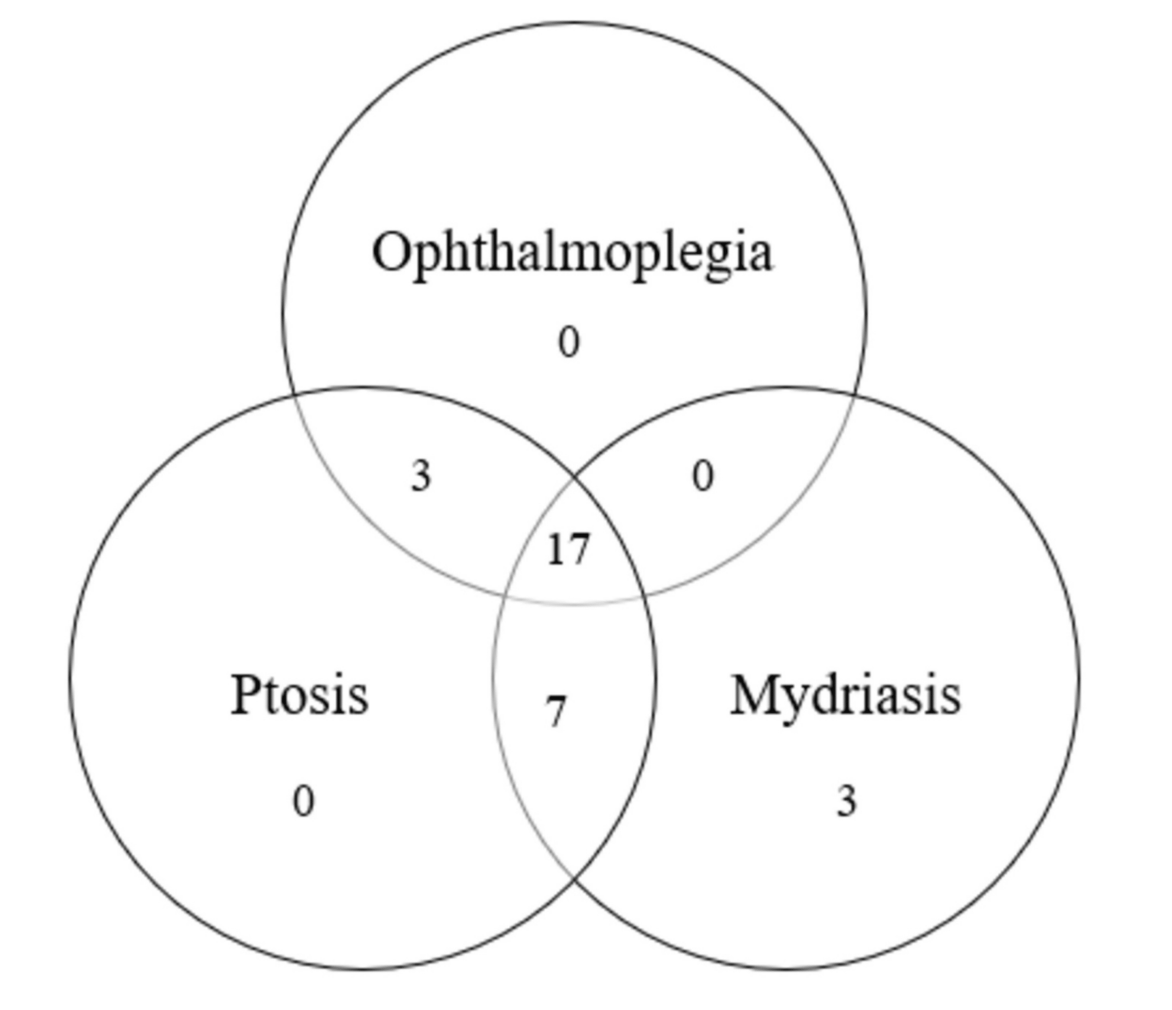
ONP symptoms present on admission ONP: oculomotor nerve palsy

At presentation, 17 (56.7%) of the patients had complete ONP. Of these, 6 had unruptured PComm aneurysms and 11 presented with SAH (Table 2). Of those with partial ONPs (n=13), the most common presentation was the combination of ptosis and mydriasis (n=7, 53.8%), with three presenting with mydriasis alone and three having ophthalmoplegia along with ptosis (Figure 1).

**Table 2:** Severity of ONP based on diagnosis of Pcomm aneurysms

	Unruptured aneurysm	Ruptured aneurysm (SAH)	Total
Complete ONP	6	11	17
Partial ONP	4	9	13
Total	10	20	30

The onset of ONP in all patients was recorded if patients could recall their symptoms or from their next-of-kin/ primary referring physician if the patients were comatose on presentation. All patients but one had documented duration of the onset of ONPs. The duration of ONP to presentation and treatment in the unruptured PComm aneurysms group was 22.4 days, and in the ruptured group was 21.1 days, which was not statistically significant (p value = 0.94). The relatively longer duration was due to patients seeking delayed medical consultation and initial misdiagnosis in the absence of other symptoms such as headaches and vomiting. Patients had a median follow-up of 107 days to assess recovery.

Postoperative recovery and outcomes

**Figure 2:**
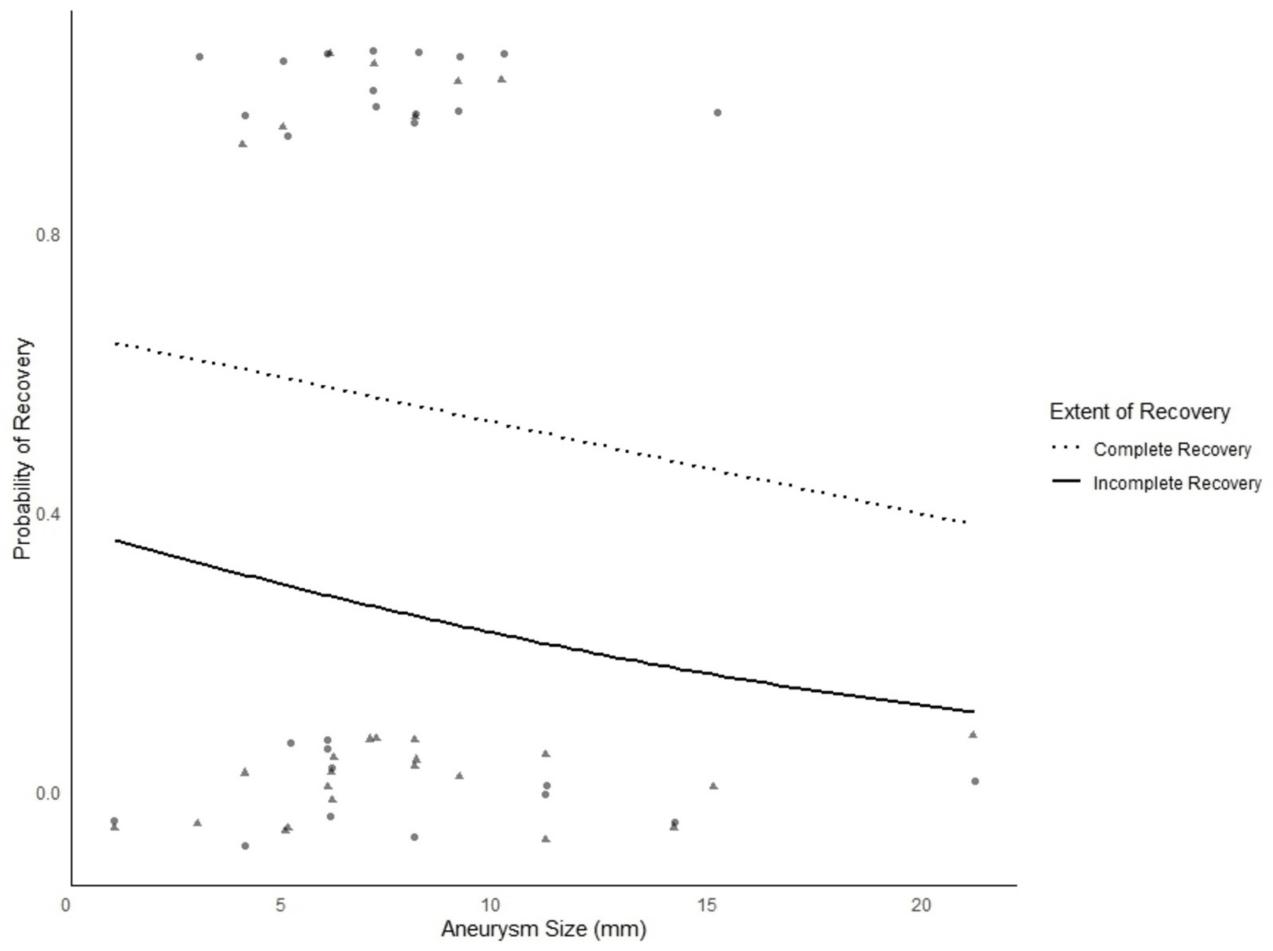
Logistic regression to understand the effect of aneurysm size on ONP recovery ONP: oculomotor nerve palsy

A total of eight (26.7%) patients had improvement in their ONP, of which five (62.5%) were from complete palsies (Table 3). Four patients had full recovery, with 50% (n=2) having presented with complete ONP and 50% (n=2) having been coiled. A logistic regression was performed to understand if the size of the aneurysm affected the recovery of ONPs (Figure 2). While there was a general negative trend (coefficient = -0.07) between the chance of improvement and increasing size of the aneurysm, this was not statistically significant, with an OR of 0.93 (0.73 - 1.19).

**Table 3:** Severity and recovery of ONP ONP: oculomotor nerve palsy

	Complete ONP at presentation	Partial ONP at presentation	Total
Improved ONP	5	3	8
Persistent ONP	12	10	22
Total	17	13	30

For patients with partial ONPs, 11 patients received coiling (84.6%, p value = 0.07), whereas there was no notable difference in treatment modality for complete ONPs (Table 4). Patients who underwent clipping had a higher rate of improvement (n=4, 40%) compared to those who underwent coiling (n=4, 20%). A logistic regression model was used across all the ruptured and unruptured cases to understand if the modality of treatment affected the recovery. In both full and partial recovery cases, there was no significant effect of treatment modality on the outcome. The OR of full recovery with endovascular treatment was 0.20 (0.03-1.07) when compared to microsurgical clipping. The OR for partial improvement of ONP with endovascular treatment was 0.38 (0.07 - 2.04).

**Table 4:** Severity and recovery of ONPs based on treatment modalities ONP: oculomotor nerve palsy

	Clipping	Coiling	Total
Complete	8	9	17
Partial	2	11	13
Total	10	20	30
Improved	4	4	8
Persisted	6	16	22
Total	10	20	30

There was no difference in ONP improvement between patients with unruptured and ruptured aneurysms, which could be attributed to a small cohort of 10 patients (Table 5). The median days to full recovery of ONP was 30.5 days. The time from onset to presentation of ONPs had no difference in the recovery of ONPs. The mean length of stay postoperatively was 13.1 ± 11.8 days, with no significant difference in length of stay between the two treatment modalities (clipping - 12 ± 11.7 days, coiling - 14 ± 12.4 days, p value = 0.69). Four patients did not survive the initial admission, of which three had undergone endovascular treatment and one had undergone surgical clipping. The ONPs of these patients persisted at the time of death and they could not be followed up to assess their improvement; hence, they were included in the persisted group, despite potential attrition bias. 

**Table 5:** Recovery of ONPs based on diagnosis of Pcomm aneurysm ONP: oculomotor nerve palsy; Pcomm: posterior communicating artery

	Improved ONP	Persistent ONP	Total
Unruptured aneurysm	4	6	10
Ruptured aneurysm (SAH)	4	16	20
Total	8	22	30

## Discussion

Recovery of ONPs after treatment of PComm aneurysms continues to be a topic of interest across many studies, with variable results. Some studies found that the majority of patients show some degree of improvement after clipping, with the recovery period lasting from 90 days to 12 months [14,17]. Others found that endovascular coiling was the most effective treatment for full recovery of ONP. Intuitively and frequently cited, microsurgical clipping is favourable in improving the recovery trajectory of ONPs as it allows direct decompression of the third nerve by alleviation of the mass effect from the aneurysmal sac. However, the lack of improvement of ONP in the microsurgical cohort is often attributed to persistent irritation by subarachnoid blood and direct mechanical compression of the third nerve by the surgical clip [18]. Along similar considerations, as endovascular treatment results in thrombosis and persistent compression from the coil mass, recovery is often slower or minimal [11]. Endovascular versus surgical treatment for improvement of oculomotor nerve palsy caused by unruptured posterior communicating artery aneurysms [19]. On the other hand, due to the nature of the endovascular procedure, it is unlikely to be an iatrogenic injury to the nerve, allowing time for recovery. Persistence of ONP despite either treatment is likely due to sustained, irreversible ischaemic damage to the endoneurial sheath [20]. The results remain conflicting in the literature. Small cohorts, choice of treatment modality, anatomical consideration, and presence of other co-morbidities are some of the reasons that could be contributing to the vast differences across studies. 

Our neurosurgical centre published a case series of 18 patients in 2014 [16]. The group treated by endovascular means was significantly older; however, there was no statistical difference in the improvement of ONPs between the two groups. The lack of difference was attributed to overall better coiling results rather than inferior clipping results. Given the overall small numbers, the authors proposed that larger consecutive studies may be required to address this. As the choice of treatment modality depends on a significant number of factors, such as aneurysm size and morphology, patient age, and co-morbidities, etc., it would be difficult to conduct a prospective study in which the treatment modality is dependent on ONPs. Our current study of 30 patients continues to demonstrate similar results. Older patients are likely to receive endovascular treatment due to concerns of surgical morbidity and mortality, and this was consistent in our data, with coiling being favoured in the older age group. Although regression analysis showed that the endovascular treatment was less likely to achieve ONP recovery, this was not statistically significantly different from the microsurgical clipping. Despite a larger number in the current series, the cohort still remains too small to demonstrate the significant difference seen in the study by Gaberel et al. [10].

A study by Jha et al. found that the rate of ONP recovery was better in patients with unruptured aneurysms than SAH [21]. Unruptured PComm aneurysms have very similar morphologic and haemodynamic characteristics to SAH [22]. In our cohort, 40% of patients in the unruptured group recovered as compared to 20% in the ruptured group. However, the numbers were too small to reach statistical significance; hence, it is difficult to determine if ONPs from unruptured aneurysms have better recovery rates based on our limited data. However, from a pathophysiological perspective, this would be in keeping with the lack of irritation due to SAH and the presence of ONP secondary to direct compression and the pulsatile nature of an aneurysm.

Complete ONP was more common in our cohort than partial ONPs (17 versus 13). 90% of our patients had pupillary involvement, which is expected as the pupillomotor fibres of the oculomotor nerve course along the superficial aspect of the nerve [2,4,17] near the PComm artery. In our cohort, 26.7% had improvement of their ONPs. Studies that have shown improvement in ONPs have demonstrated that an initial partial ONP is more likely to result in full recovery after treatment [23]. Our study had no statistical difference when the initial presenting ONP was considered, which again could be attributed to the small number of patients. The impact of the COVID-19 pandemic on face-to-face assessment worldwide [24] also affected our outpatient clinics, resulting in a lack of proper ophthalmological assessment, and the study, depending on patient/non-specialist assessment of the recovery, may have resulted in a lower number of patients having a documented recovery of their ONPs.

Despite our current study showing some favourable outcomes in the clipping cohort as compared to the coiling cohort, these results may vary across centres and patient populations. Safe treatment of the aneurysm will always remain a priority, with ONP recovery being an added benefit. Future advancements in endovascular treatment methods, such as stents or improved microsurgical techniques, such as endoscopes, could ensure that the oculomotor nerve has been decompressed adequately and change future outcomes. Based on the results of our previous [16] and the current study, it would be difficult to adequately counsel patients and their families with regard to the expected recovery outcomes associated with ONP.

The limitations of this study are that it was retrospective in nature and had a small number of patients, as also seen in previous studies. Furthermore, the impact of the COVID-19 pandemic limited face-to-face follow-up and examination of patients; hence, it introduced potential bias into the outcome measurement. 

## Conclusions

There was no significant difference found in rates of recovery of ONPs between surgical clipping and endovascular coiling, although the findings of this study are limited by the small sample size. Both modalities continue to appear equally effective for aneurysm management. As the choice of treatment modality will continue to be dictated by a number of clinical and anatomical factors, rather than solely ONP recovery, it is difficult to ascertain the superiority of one over the other; thus, the literature will likely continue to have conflicting results. 

## References

[1] He W, Hauptman J, Pasupuleti L (2010). True posterior communicating artery aneurysms: are they more prone to rupture? A biomorphometric analysis. J Neurosurg.

[2] Golshani K, Ferrell A, Zomorodi A, Smith TP, Britz GW (2010). A review of the management of posterior communicating artery aneurysms in the modern era. Surg Neurol Int.

[3] Donaldson L, Edington A, Vlok R (2022). The incidence of cerebral arterial vasospasm following aneurysmal subarachnoid haemorrhage: a systematic review and meta-analysis. Neuroradiology.

[4] Kim JY, Choi SC (2014). Third cranial nerve palsy and posterior communicating artery aneurysm. Clin Exp Emerg Med.

[5] Haider AS, Gottlich C, Sumdani H, Layton KF, Doughty K (2019). Acute oculomotor nerve palsy caused by compression from an aberrant posterior communicating artery. Cureus.

[6] Debnam JM, Guha-Thakurta N Retrochiasmatic optic pathway. Imaging Atlas of Ophthalmic Tumors and Diseases.

[7] Nam KH, Choi CH, Lee JI, Ko JG, Lee TH, Lee SW (2010). Unruptured intracranial aneurysms with oculomotor nerve palsy : clinical outcome between surgical clipping and coil embolization. J Korean Neurosurg Soc.

[8] Khizar A, Umer AW (2024). Unilateral pupil sparing oculomotor nerve paresis with an anterior communicating artery aneurysm: a case report with literature review. Brain Hemorrhage.

[9] Yanovitch T, Buckley E (2007). Diagnosis and management of third nerve palsy. Curr Opin Ophthalmol.

[10] Gaberel T, Borha A, di Palma C, Emery E (2016). Clipping versus coiling in the management of posterior communicating artery aneurysms with third nerve palsy: a systematic review and meta-analysis. World Neurosurg.

[11] Sheehan MJ, Dunne R, Thornton J, Brennan P, Looby S, O'Hare A (2015). Endovascular repair of posterior communicating artery aneurysms, associated with oculomotor nerve palsy: a review of nerve recovery. Interv Neuroradiol.

[12] Signorelli F, Pop R, Ganau M (2020). Endovascular versus surgical treatment for improvement of oculomotor nerve palsy caused by unruptured posterior communicating artery aneurysms. J Neurointerv Surg.

[13] Tabata S, Take Y, Kimura T (2024). Recovery of oculomotor nerve palsy after surgical and endovascular repair of unruptured internal carotid-posterior communicating artery aneurysms. World Neurosurg.

[14] Tian LQ, Fu QX (2020). Recovery of posterior communicating artery aneurysm induced oculomotor nerve palsy: a comparison between surgical clipping and endovascular embolization. BMC Neurol.

[15] Kassis SZ, Jouanneau E, Tahon FB, Salkine F, Perrin G, Turjman F (2010). Recovery of third nerve palsy after endovascular treatment of posterior communicating artery aneurysms. World Neurosurg.

[16] Patel K, Guilfoyle MR, Bulters DO (2014). Recovery of oculomotor nerve palsy secondary to posterior communicating artery aneurysms. Br J Neurosurg.

[17] da Costa MD, Lima JV, Zanini MA (2023). Risks for oculomotor nerve palsy and time to recovery after surgical clipping of posterior communicating artery aneurysms: a multicenter retrospective cohort study. Neurosurgery.

[18] Kim E (2020). Clip compression injury of the oculomotor nerve: its prevention and recovery. Korean J Neurotrauma.

[19] Scerrati A, Trevisi G, Sturiale CL (2021). Radiological outcomes for endovascular treatment of posterior communicating artery aneurysms: a retrospective multicenter study of the occlusion rate. J Integr Neurosci.

[20] Modi P, Arsiwalla T (2023). Cranial nerve III palsy. StatPearls [Internet].

[21] Jha VC, Sinha V, Abhijit V, Jha N, Singh SK (2024). Comparative analysis of the risk factors influencing recovery of function from oculomotor nerve palsy in unruptured and ruptured posterior communicating artery aneurysms. Turk Neurosurg.

[22] Yu Y, Xu J, Fang Y (2013). Analysis of morphologic and hemodynamic parameters for unruptured posterior communicating artery aneurysms with oculomotor nerve palsy. AJNR Am J Neuroradiol.

[23] Chalouhi N, Theofanis T, Jabbour P (2013). Endovascular treatment of posterior communicating artery aneurysms with oculomotor nerve palsy: clinical outcomes and predictors of nerve recovery. AJNR Am J Neuroradiol.

[24] Byravan S, Sunmboye K (2021). The impact of the coronavirus (COVID-19) pandemic on outpatient services-an analysis of patient feedback of virtual outpatient clinics in a tertiary teaching center with a focus on musculoskeletal and rheumatology services. J Patient Exp.

